# Evaluation of limited irrigation strategies to improve water use efficiency and wheat yield in the North China Plain

**DOI:** 10.1371/journal.pone.0189989

**Published:** 2018-01-25

**Authors:** Di Zhang, Ruiqi Li, William D. Batchelor, Hui Ju, Yanming Li

**Affiliations:** 1 College of Agronomy, Hebei Agricultural University, Baoding, Hebei, China; 2 Biosystems Engineering Department, Auburn University, Auburn, AL, United States of America; 3 Institute of Environment and Sustainable Development in Agriculture, Chinese Academy of Agricultural Science, Beijing, China; Institute of Genetics and Developmental Biology Chinese Academy of Sciences, CHINA

## Abstract

The North China Plain is one of the most important grain production regions in China, but is facing serious water shortages. To achieve a balance between water use and the need for food self-sufficiency, new water efficient irrigation strategies need to be developed that balance water use with farmer net return. The Crop Environment Resource Synthesis Wheat (CERES-Wheat model) was calibrated and evaluated with two years of data which consisted of 3–4 irrigation treatments, and the model was used to investigate long-term winter wheat productivity and water use from irrigation management in the North China Plain. The calibrated model simulated accurately above-ground biomass, grain yield and evapotranspiration of winter wheat in response to irrigation management. The calibrated model was then run using weather data from 1994–2016 in order to evaluate different irrigation strategies. The simulated results using historical weather data showed that grain yield and water use was sensitive to different irrigation strategies including amounts and dates of irrigation applications. The model simulated the highest yield when irrigation was applied at jointing (T9) in normal and dry rainfall years, and gave the highest simulated yields for irrigation at double ridge (T8) in wet years. A single simulated irrigation at jointing (T9) produced yields that were 88% compared to using a double irrigation treatment at T1 and T9 in wet years, 86% of that in normal years, and 91% of that in dry years. A single irrigation at jointing or double ridge produced higher water use efficiency because it obtained higher evapotranspiration. The simulated farmer irrigation practices produced the highest yield and net income. When the cost of water was taken into account, limited irrigation was found to be more profitable based on assumptions about future water costs. In order to increase farmer income, a subsidy will likely be needed to compensate farmers for yield reductions due to water savings. These results showed that there is a cost to the farmer for water conservation, but limiting irrigation to a single irrigation at jointing would minimize impact on farmer net return in North China Plain.

## Introduction

The North China Plain (NCP) is one of the most important grain production regions in China, accounting for 56% the wheat grain production of China [1]. Annual precipitation is unevenly distributed both within and across seasons, which does not satisfy seasonal water demand for winter wheat [2]. For this reason, irrigation is required to achieve high grain yields for winter wheat in NCP. Because there are no reliable surface water sources for irrigation, groundwater is the primary source of water for irrigation. However, groundwater use exceeds the recharge rates, and the groundwater levels are dropping, causing a range of adverse environmental and economic impacts [3,4]. It was reported that one way to alleviate groundwater depletion is to decrease sown area of winter wheat and irrigation amount [3,5]. However, this would threaten national food security if winter wheat production was significantly reduced in NCP [6]. The scale of the problem is such that it is now imperative to find ways to reduce agricultural water use without affecting food supplies [7]. To achieve the trade-offs between alleviating water resource over-exploitation and food self-sufficiency, a greater adoption of water-efficient production systems appears to be the best solution [8,9].

In agricultural production in NCP, farmers usually irrigate three to five times via flood irrigation over the winter wheat growing season. Previous studies have shown that double irrigation, one at jointing and the second at heading or anthesis results in higher water use efficiency and yield [10]. Nevertheless, groundwater use is not sustainable even under optimized irrigation regimes. To stabilize the groundwater table, new irrigation strategies must be developed that will maintain yield and increase water use efficiency (WUE) [11].

Reducing the frequency of irrigations and the amount of water applied would help alleviate groundwater depletion. This strategy is referred to as deficit irrigation. Deficit irrigation is defined as the application of water below full crop water requirements. Moderate water deficit during grain filling increases mobilization of assimilate stored in vegetative tissues and grain, resulting in greater grain yield and WUE. Supplemental irrigation at early growth stages enables roots to grow deeper into soil layers, which enhances uptake of soil-stored water from the deeper subsoil layers [12]. Thus, deficit irrigation promotes vegetative growth and more efficient soil water use during the reproductive stage [13]. Recommendations for limited irrigation differ. Some research reported that irrigation applied before planting under limiting condition increases yield [14,15]. Other studies suggest that one irrigation at jointing can increase grain yield and maximize WUE [11,12]. Some studies have tested irrigation strategies based on pan evaporation or monitoring soil water levels. These strategies recommended irrigation amounts of 50–100 mm divided into applications of 20–40 mm each [16,17]. However, in practice it is still difficult for farmers to use such criteria. Thus it is important to clarify how to schedule supplemental irrigation to minimize yield loss under a limited water supply.

Agricultural systems modeling has proven to be an effective tool to investigate the potential impacts of climate variability and management strategies on crop productivity, resource use efficiencies and environmental impacts of farming systems [18,19]. In recent years, the Decision Support System for Agrotechnology Transfer (DSSAT) model [20,21] has been successfully used to analyze irrigation strategies for wheat in China [22–28]. However, these studies did not evaluate how the yield of winter wheat and soil water balances are affected by water limiting irrigation strategies to conserve water over the long term.

Water pricing plays a significant role in coordinating water use and benefits increases [29]. In the NCP, irrigation water is currently free, and farmers currently only pay the power costs associated with pumping groundwater [30]. Thus farmers are not charged volumetric prices and so are not encouraged to conserve water [31]. In China, much research has been conducted to assess water rights and irrigation price policy [32–36]. However, there is limited research available to evaluate the impact of potential water cost policies on crop productivity under deficit irrigation strategies.

The objectives of this study were: 1) to calibrate the DSSAT-CERES-Wheat model to accurately simulate winter wheat growth and yield in the North China Plain under limiting irrigation condition; 2) to simulate winter wheat grain and biomass yields, evapotranspiration, and water use efficiency responses to different irrigation scheduling practices using long-term weather data; 3) evaluate the impact of water price on economic return under different irrigation strategies.

## Materials and methods

### Study site

**Fig 1:**
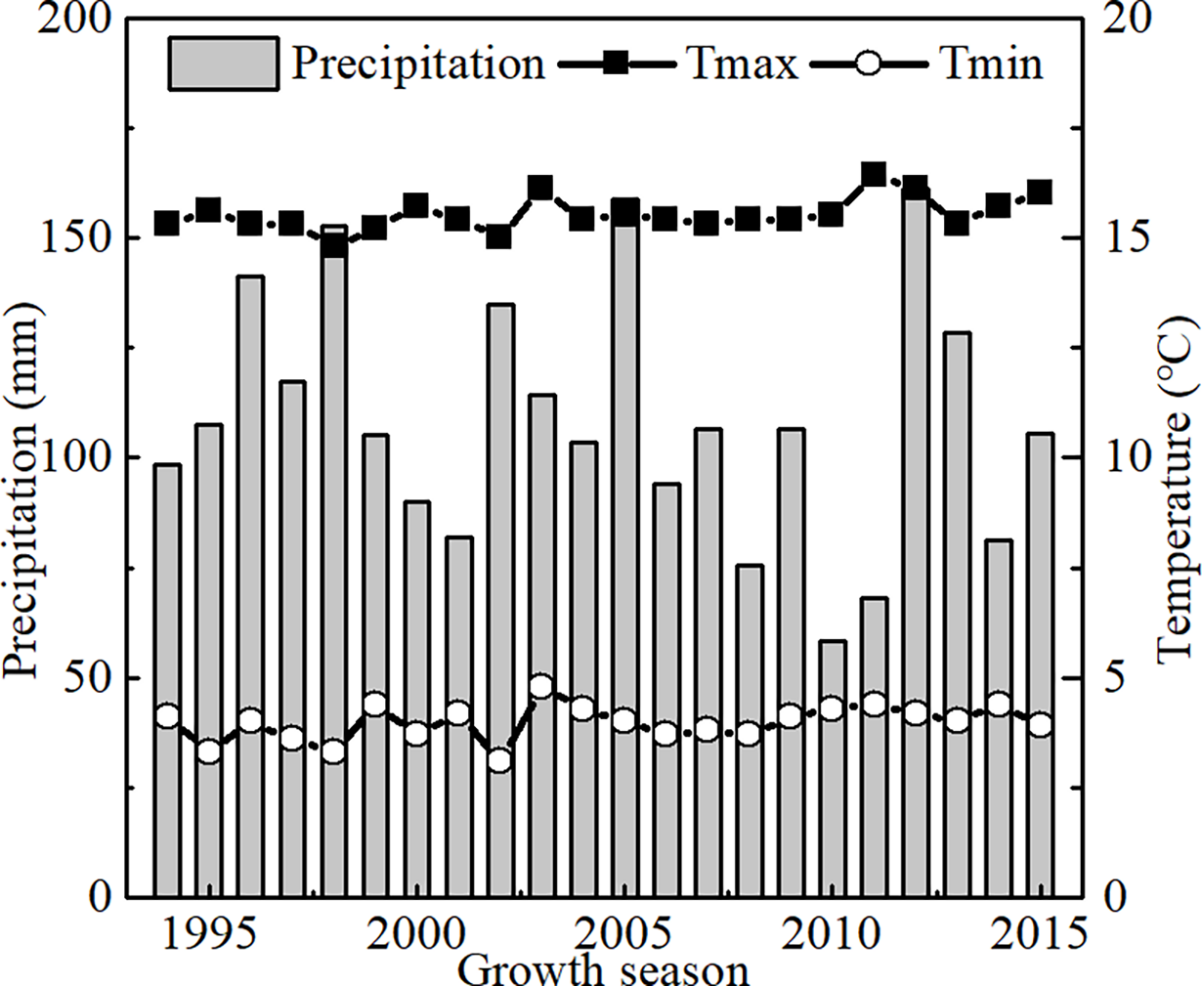
Distribution of precipitation, maximum (Tmax) and minimum temperature (Tmin) at Gaocheng, over 22 growth season.

The study was carried out in Gaocheng District, Shijiazhuang City, Hebei Province, China, which is in the middle of NCP. The typical cropping system is a winter wheat and summer maize double cropping system. The area is semi-arid with a monsoon climate, with average annual temperature of 11–14°C. The average precipitation of winter wheat is 108.7 mm form 1994 to 2015 (Fig 1). More than 70% of the precipitation occurs from July to September [37]. Only 20–30% of the precipitation occurs from October to early June during the wheat growing season. The soil type was clay-loam.

### Field management and experiment data

Field experiments were conducted in 2013–2014 (experiment 1) and 2014–2015 (experiment 2). Experiment 1 (Table 1) consisted of three irrigation treatments, including normal farmer practice (100 mm applied at jointing and 100 mm at anthesis with 240 N kg ha^-1^), deficit irrigation level 1 (60 mm applied at jointing and 60 mm at anthesis with 195 N kg ha^-1^), deficit irrigation level 2 (45 mm applied at jointing and 30 mm applied at anthesis and 30 mm applied at grain filling with 195 N kg ha^-1^). Experiment 2 was set up as a split-plot design with four irrigation treatments in the main plots and three nitrogen levels in the sub-plots. A local winter wheat cultivar, Shimai 18 was planted, and the plot size was 7 m × 10 m with three replications in all experiments. In experiment 1, the winter wheat was sown on October 8 with a plant density of 300 plants m^-2^. In experiment 2, winter wheat was sown on October 9 with a plant density of 317 plants m^-2^. Weeds, insect pests, and diseases were properly controlled and the crops were not limited by other nutrients. Thirty plants were selected to determine the above ground dry matter accumulation at pre-winter, double-ridge, jointing, booting, anthesis, grain filling and maturity in all experiments. The leaf area index (LAI) was determined from the same samples as used for dry matter weight. Leaf area was calculated as leaf length × leaf width × 0.83. All plant samples were oven dried at 105°C for 30 min, then dried at 75°C for 72 h until reaching constant weight. At maturity, 2 m^2^ of plants in each plot was harvested for the determination of grain yield.

**Table 1:** Water and nitrogen treatments in the experiments conducted at Gaocheng, China (2013–2015).

	Total irrigation amount (mm)	Jointing Irrigation (mm)	Anthesis Irrigation (mm)	Grain Filling Irrigation (mm)	N application (N kg ha^-1^)
Experiment 1	200	100	100	0	240
	120	60	60	0	195
	105	45	30	30	195
Experiment 2	67.5	67.5	0	0	120, 180, 240
	67.5	37.5	30	0	120, 180, 240
	67.5	37.5	15	15	120, 180, 240
	135	67.5	67.5	0	120, 180, 240

### CERES-Wheat model calibration and evaluation

The DSSAT-CERES-Wheat model version 4.6[20,21] was used to simulate above-ground biomass, grain yield, and evapotranspiration at the study site. The model requires daily weather data, soil profile characteristics, crop management data, and genotype coefficients as general inputs. Daily weather data from 1994 to 2016 including daily average, maximum and minimum temperature, precipitation, and sunshine hours were obtained from the weather station located in Gaocheng, China. Daily solar radiation was calculated using daily sunshine hours using the Weatherman software provided with DSSAT 4.6. The data of Yang et al. [22] were used to establish soil profile characteristics including lower limit, drained upper limit and saturated water holding capacity. The cultivar coefficients required to run the DSSAT-Wheat model were calibrated with field observations of biomass obtained in the 2013–2014 experiments. The cultivar coefficients required to simulate crop growth are shown in Table 2. For the remaining growth and yield coefficients, an iterative procedure was used to obtain a close match between simulated and measured leaf area index (LAI), biomass and grain yield. Following calibration, the model was evaluated using field experiment 2. To quantify the goodness of fit of the model, two common statistical indicators were used: root mean square errors (RMSE), and coefficient of determination (R^2^).

$$RMSE=\sqrt{\frac{\sum_{i=1}^{n}{\left(S_{i}-M_{i}\right)}^{2}}{n}}$$

$$R^{2}=\frac{{\left[\sum_{i=1}^{n}\left(M_{i}-M_{avg}\right)(S_{i}-S_{avg})\right]}^{2}}{\sum_{i=1}^{n}{\left(M_{i}-M_{avg}\right)}^{2}\sum_{i=1}^{n}{\left(S_{i}-S_{avg}\right)}^{2}}$$

Where *M*_*i*_ and *S*_*i*_ are the observed and simulated values, *M*_*avg*_ and *S*_*avg*_ are the mean observed and simulated values, respectively; *n* is the number of samples.

**Table 2:** Genetic coefficients calibrated for the 2013–2014 and 2014–2015 seasons.

Genetic coefficient	Definition	Calibrated values
P1V	Days, optimum vernalizing temperature, required for vernalization	45
P1D	Photoperiod response (% reduction in rate/10 h drop in pp)	68
P5	Grain filling (excluding lag) phase duration (°C day)	800
G1	Kernel number per unit canopy weight at anthesis (g)	31
G2	Standard kernel size under optimum conditions (mg)	35
G3	Standard, non-stressed mature tiller weight (including grain) (g dry weight)	1.4
PHINT	Interval between successive leaf tip appearances (°C day)	100

### Evaluation of long-term irrigation strategies

The genetic coefficients calibrated (2013 and 2014) and evaluated (2014 and 2015) were used in the DSSAT-CERES-Wheat model (Verson 4.6) to simulate winter wheat biomass and grain yield, evapotranspiration and water use efficiency in response to different irrigation schedules using historical weather data from 1994–2016. The irrigation schedules that were evaluated are shown in Table 3. The fertilizer strategy used in all simulations was 120 N kg ha^-1^, 150 P_2_O_5_ kg ha^-1^, 100 K_2_O kg ha^-1^ applied at sowing and 120 N kg ha^-1^ was applied at jointing. Since inter-annual variation of precipitation varies strongly in local areas, the rain fall datasets were classified into three categories. Wet year (n = 6), normal year (n = 9) and dry year (n = 7) were years with percentage of deviation of averages above 10%, between -10% and 10%, and below -10%, respectively [38].

**Table 3:** Irrigation schedules simulated with the CERES-Wheat model.

Treatment	Sowing, mm	Double ridge, mm	Jointing, mm	Anthesis, mm	Grain filling, mm	Total, mm
T1			70	70		140
T2			30	30		60
T3			30	30	30	90
T4		30	30	30		90
T5		30	30	30	30	120
T6			70	30		100
T7	70					70
T8		70				70
T9			70			70
T10				70		70

Water use efficiency was calculated as the ratio of grain yield (kg ha^-1^) to evapotranspiration (mm). Net water use was described by consumption of groundwater. The meaning of a negative use is that storage in the aquifer has been dropping. The net water use was calculated using the following equation [39]:

NWU=D−I

Where, *D* = groundwater recharge, mm and *I* = irrigation pumping from groundwater, mm.

The marginal net return (MNR, $ ha^-1^) and relative marginal net return (RMNR, $ mm^-1^ irrigation water) were computed for each scenario by the following equations:

$$MNR=(Y_{I}-Y_{0})\times Price-Cost$$

$$RMNR=\frac{MNR}{irrigation\;amount}$$

Where *Y_I_* (kg ha^-1^) is the grain yield from treatments with irrigation, *Y*_0_ (kg ha^-1^) is the grain yield from treatment without irrigation, Price is the wheat grain price of 0.36 $ kg^-1^ and Cost is the sum of all fixed costs ($ ha^-1^).

## Results

### Model calibration and evaluation

**Fig 2:**
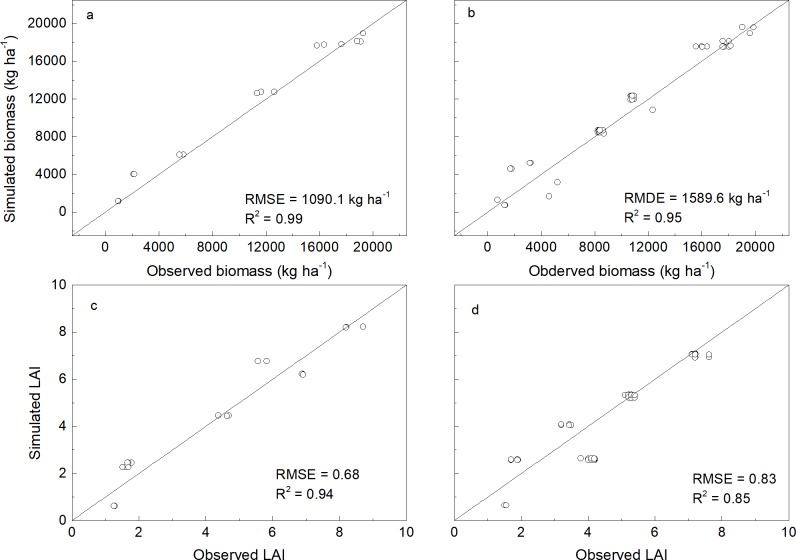
Comparisons of measured and observed above-ground biomass (a for 2013–2014 and b for 2014–2015) and LAI (c for 2013–2014 and d for 2014–2015). a and c were used to calibrate the model, b and d were used to validate the model.

The genetic coefficients for the CERES-Wheat model were calibrated to accurately simulate winter wheat growth and yield response to irrigation management experiments conducted in the North China Plain. The genetic coefficients for winter wheat that were obtained by fitting the model simulation to observed biomass data are shown in Table 2. The data for 2013–2014 were chosen to calibrate the model (Fig 2A and 2C). Biomass simulations of winter wheat agreed well with observations with an R^2^ of 0.99 and RMSE of 1090.1 kg ha^-1^ across different irrigation treatments. The simulated leaf area index also agreed well with observed measurements with an R^2^ of 0.94 and RMSE of 0.68 m^2^ m^-2^. Thus, the model did an excellent job in describing the yield response to different irrigation treatments for the calibration and evaluation treatments and years.

**Fig 3:**
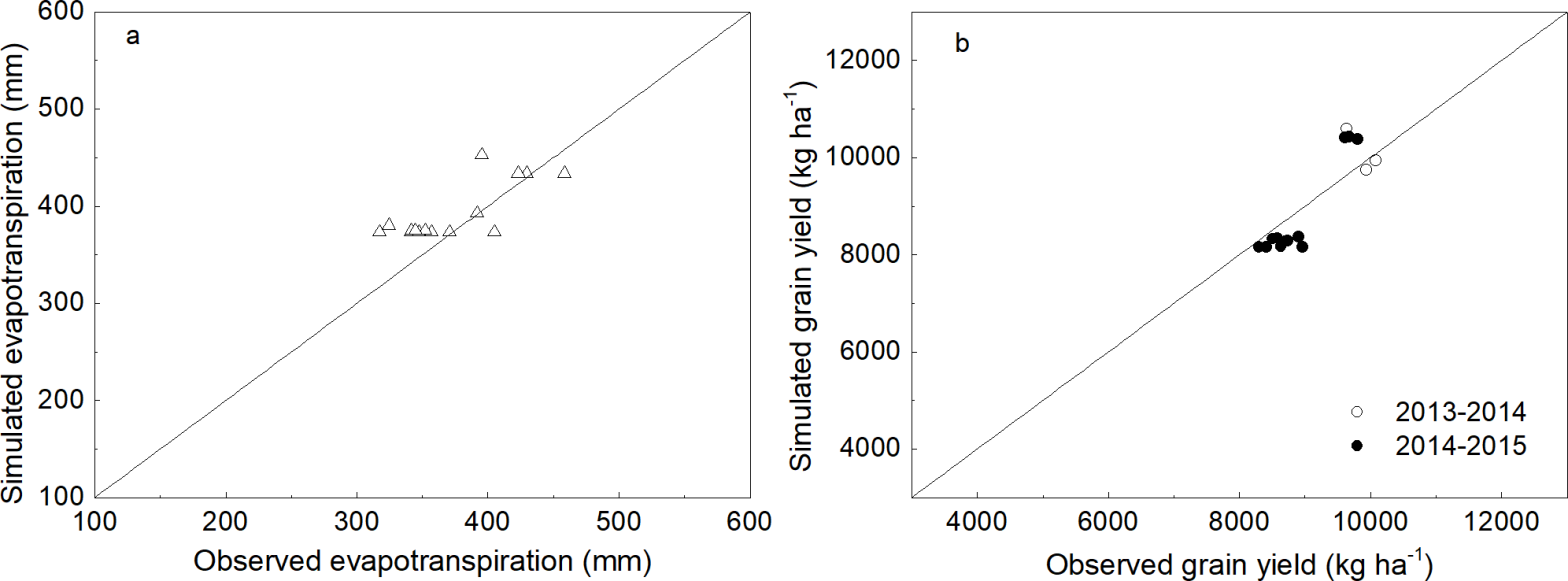
Validation results of the CERES-wheat model for evapotranspiration (a) and grain yield (b).

Once calibrated, the model was evaluated for LAI and above-ground biomass in 2014–2015, and grain yields and evapotranspiration in 2013–2015. The model gave a good prediction of the grain yield and evapotranspiration (ET) with an R^2^ of 0.82 and an RMSE value of 529.1 kg ha^-1^ and 32.5 mm of ET, respectively (Fig 3A and 3B). In addition, simulated above-ground biomass agreed well with observations with an R^2^ of 0.95 and 1589.6 kg ha^-1^. Acceptable performance of the LAI prediction was indicated by the R^2^ of 0.85 and RMSE of 0.83 m^2^ m^-2^. These results indicated that the model was able to accurately simulate above-ground biomass, grain yield and evapotranspiration of winter wheat in response to different irrigation management treatments in the North China Plains.

### Grain and biomass yield in response to different irrigation strategies and precipitation amounts

**Fig 4:**
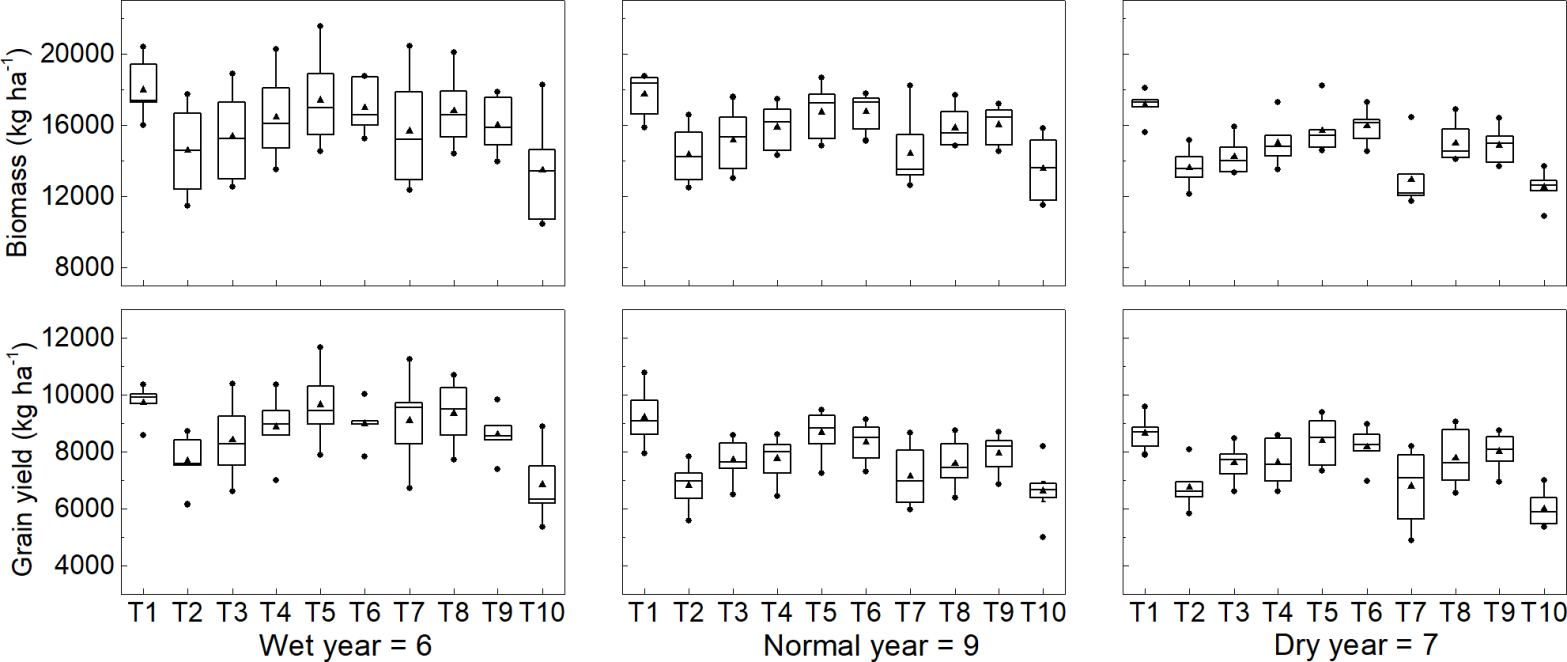
Simulated grain and biomass yield for different irrigation treatments. The triangle indicates the median. The circle indicates the 75 and 25 percent. The middle line indicates 50 percent.

**Fig 5:**
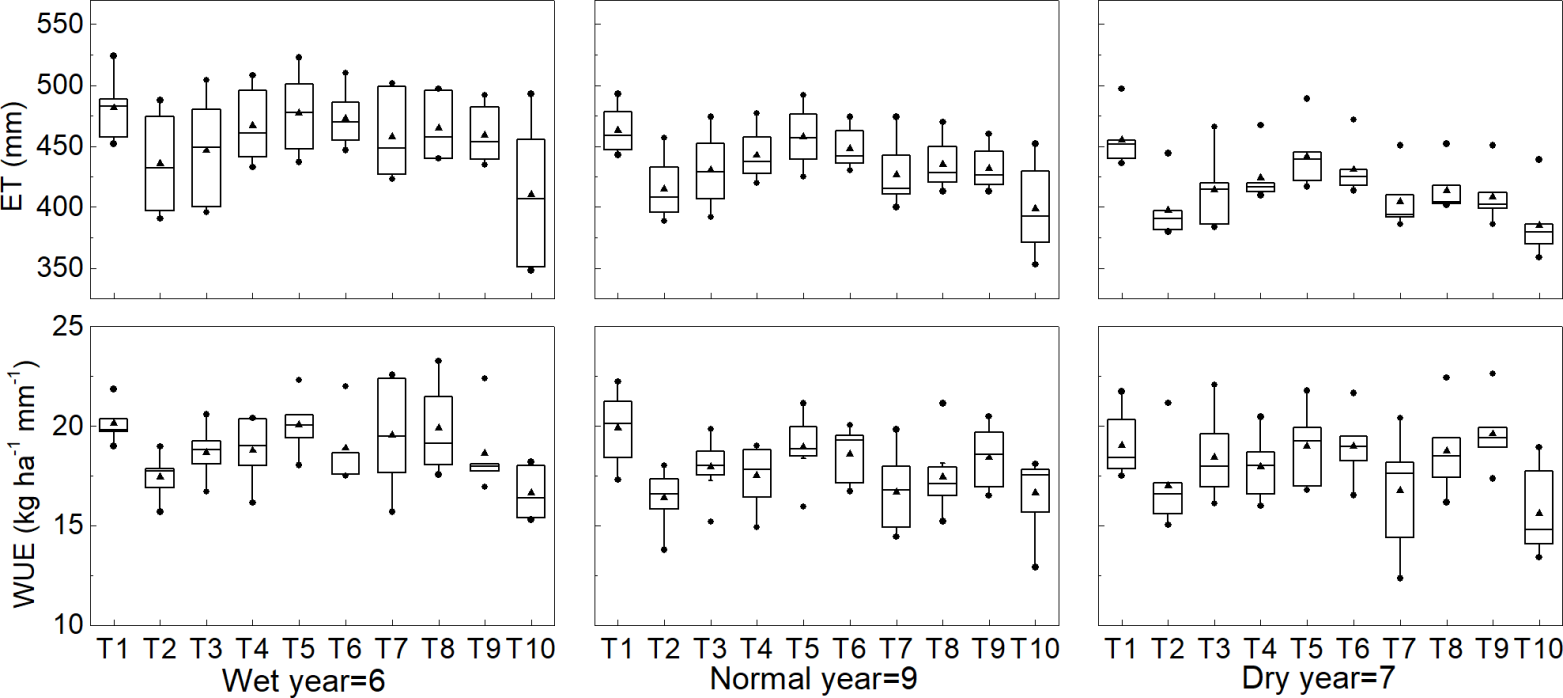
Simulated evapotranspiration (ET) and water use efficiency (WUE) for different treatments. The triangle indicates the median. The circle indicates the 75 and 25 percent. The middle line indicates 50 percent.

The calibrated and evaluated CERES-Wheat model was used to investigate winter wheat biomass and grain yield, evapotranspiration, and WUE responses to different irrigation scheduling practices using historical weather data from 1994–2016 for the North China Plain (Figs 4 and 5). Simulated grain yield was significantly affected by the timing and amount of irrigation across the simulation years (Fig 4). Simulated double irrigation at jointing and anthesis (T1) gave the highest simulated yield for wet, normal and dry years. Four irrigations (T5) and double irrigation (T1) gave no differences in simulated yield. Single irrigation at jointing (T9), with half of the irrigation amount of the double irrigation treatment (T1), gave 88% of the simulated yield for the double irrigation treatment (T1) grain yield in wet years, 86% of that in normal years, and 91% of that in dry years. Compared with reduced irrigation at jointing and anthesis (T2), a single irrigation at jointing (T9) increased yield by 13% in wet years, 15% in normal years, and 19% in dry years. A single irrigation at jointing (T9, 70 mm) produced higher simulated grain yield than triple irrigation treatments (T3 and T4, 90 mm) in normal and dry years. The highest simulated yield was found for irrigation at jointing (T9) in normal years and dry years, and for irrigation at the double ridge stage (T8) in wet years. Overall, this indicates that it is better to irrigate at critical stages rather than irrigate multiple times in low volumes. A single irrigation during early spring (double ridges or jointing) produced simulated yields that were not significantly different from double irrigation.

Similar to grain yield, simulated biomass yield was significantly affected by the timing and amount of irrigation across the simulation years. Single irrigation at jointing (T9) produced approximately 89% of the biomass yield compared to double irrigation (T1) across the simulation years, which is equal with that of triple irrigation at double ridge and jointing and anthesis (T4), and produced little higher biomass yield than triple irrigation at jointing and anthesis and grain filling (T3). Moreover, a single irrigation at earlier spring growth stages (T8 and T9) produced higher biomass yield than single irrigation at sowing (T7) or anthesis (T10). It is suggested that single irrigation at earlier spring growth stages (double ridge or jointing) may be the optimal measures.

### Evapotranspiration and water use efficiency in response to different irrigation and precipitation

Evapotranspiration and water use efficiency for different years and irrigation treatments are shown in Fig 5. Simulated evapotranspiration reduced with the decreasing irrigation amount over the 22 year simulation period. Evapotranspiration also was affected by the timing of irrigation across the simulation years. Compared to the other single irrigation treatments, single irrigation at anthesis (T10) reduced the evapotranspiration over the simulation period. While deficit irrigation at jointing (T9) and that at double ridge (T8) exhibited no differences. When come to water use efficiency, it not only depended on irrigation amount and timing, but also on precipitation. The maximum mean water use efficiency was obtained for irrigation at jointing (T9) in dry years, followed by double irrigation treatment (T1) and four times irrigation (T5). While in normal years double irrigation resulted in the highest water use efficiency, followed by four times irrigation (T5) and single irrigation at jointing (T9). Under the condition of single irrigation in wet years, the water use efficiency was higher for single irrigation at double ridge (T8) than that for single irrigation at jointing (T9). The water use efficiency for double irrigation (T1) was not significantly different from that of single irrigation at jointing (T9) in wet years and normal years. Hence, during the winter wheat growing season, single irrigation should be applied at earlier spring growth stages (double ridge and jointing) to save water and to obtain high WUE and grain yield.

**Fig 6:**
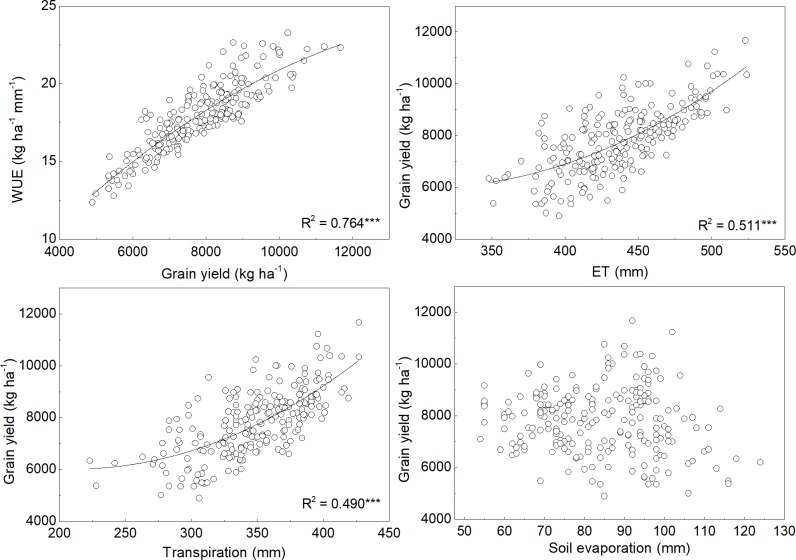
Grain yield response to evapotranspiration (ET) and transpiration and soil evaporation over 22 years.

Fig 6 showed the water use efficiency response to grain yield, and grain yield response to evapotranspiration and transpiration and soil evaporation across simulation years. The response of water use efficiency to grain yield can be described using a quadratic equation (R^2^ = 0.764, *P*<0.01). The relationships indicated the importance of obtaining relatively high yield for attaining high WUE. The relationships between grain yield and evapotranspiration or transpiration were described by a quadratic equation. However, there was no relation between grain yield and soil evaporation. That suggested that attaining high grain yield should maintain high evapotranspiration, especially transpiration.

### Economic analysis

In traditional cultivation systems of high-yielding wheat, it was usual to irrigate more than 3 to 5 times during wheat growing seasons. So three times irrigation (pre-winter, jointing and anthesis) was selected as farmer irrigation with more sowing seeds, which was simulated over 22 years in this study. In the North China Plain the water is free. In this study the water price is power cost of pumping groundwater. Grain yield and economic costs for different irrigation regimes were shown in Table 4. With the increasing of irrigation amount, the yield and net income increased, but the net water use decreased. The farmer irrigation produced the highest yield and net income. Compared with farmer irrigation, recommending irrigation reduced grain yield by 5.25%, but the net income only decreased by 7.73% because of irrigation and seed costs decreasing. While limiting irrigation produced acceptable yield of 8244.3 kg ha^-1^, the net income of that produced 86.59% of farmer irrigation, the relative marginal return was 98.71% higher than farmer irrigation.

**Table 4:** Economic costs for different irrigation regimes over 22 seasons.

Irrigation Regime	Irrigation Amount mm	Yield kg ha^-1^	Cost $ ha^-1^	Income $ ha^-1^	MNR $ ha^-1^	RMNR $ ha^-1^ mm^-1^	NWU mm
Rainfed	0	5280.6	673	1228.0			29.4
Limiting irrigation	80	8244.3	753	2214.9	986.9	12.34	-50.6
Recommending irrigation	140	9021.1	815	2432.6	1204.6	8.60	-110.6
Farmer irrigation	210	9520.9	894	2533.5	1305.5	6.21	-180.6

US$ = 6.6 Chinese Yuan. The prices for land preparing and sowing were: water power ($0.08 m^-3^), plough ($68 ha^-1^), rotary ($45 ha^-1^), sowing wheat ($56 ha^-1^), wheat seed ($0.52 kg^-1^), herbicides and pesticides ($23 ha^-1^), harvest ($145 ha^-1^). Fertilizer prices: N ($0.59 kg^-1^), P_2_O_5_ (0.52 kg^-1^). Labor price: $8 day^-1^.

**Fig 7:**
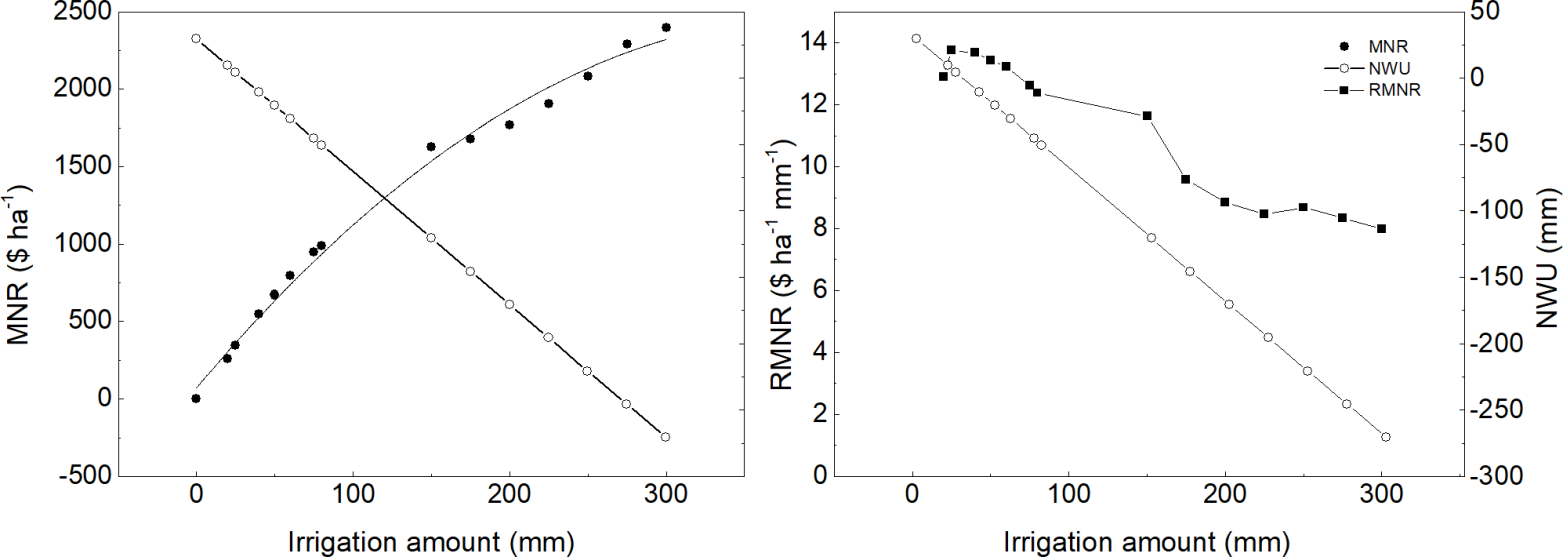
Simulated marginal net return (MNR), relative marginal net return (RMNR) and net water use (NWU) for different irrigation over 22 year seasons.

**Fig 8:**
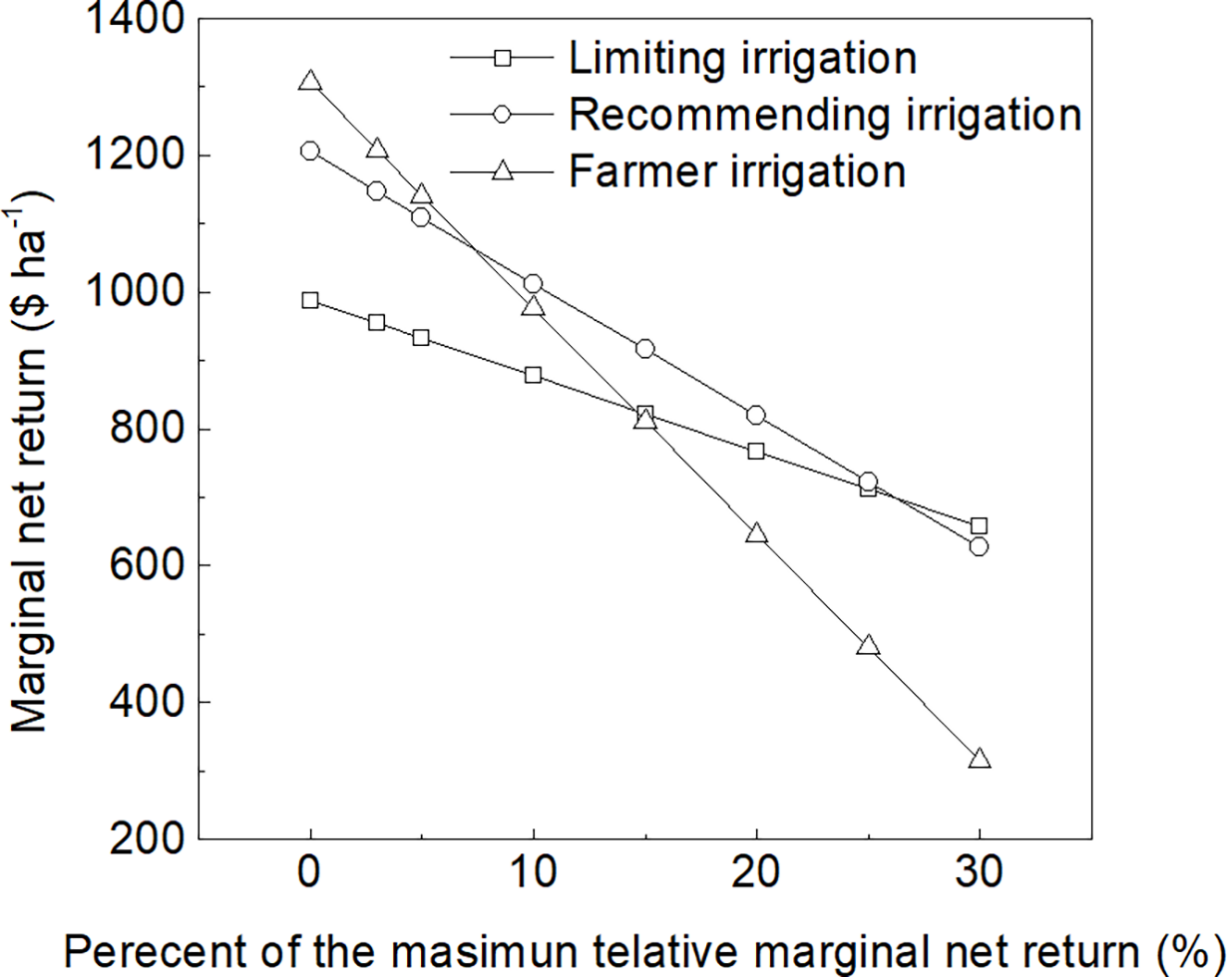
Simulated net return for different irrigation strategies over 22 seasons.

Marginal net return, net water use and relative marginal net return in response to irrigation amount were shown in Fig 7. With the increasing of irrigation amount, the marginal net return increased, but the relative marginal net return and net water use decreased. The maximum relative marginal net return was 13.77 $ ha^-1^ mm^-1^. Take the maximum relative marginal net return as the standard rate, a range of water price rate was set out. So the cost of water was the sum of water power cost and water price rate which is the percentage of the maximum relative marginal net return. The marginal net return in response to water price rate was shown in Fig 8 under different irrigation strategies. The marginal net return was reduced as the rate of irrigation water price increased. The slope of farmer irrigation was higher than others, because of much more irrigation amount than others. When the rate was 7.33% of the maximum relative marginal net return, the marginal net return between recommending irrigation and farmer irrigation were equal, and both were higher than limiting irrigation. As water price rate was lower than 7.33%, the marginal net return of farmer irrigation was lower than that of recommending irrigation. The marginal net return of farmer irrigation was not lower than that of limiting irrigation until the rate increased to above 14.47%. At the rate of 26.4%, the marginal net return of both limiting irrigation and recommending irrigation was 696.5 $ ha^-1^. The recommending irrigation produced lower marginal net return than limiting irrigation as the rate exceeds 26.4%.

## Discussion

Extensive studies showed that once the model was properly calibrated, it was able to predict the biomass growth, grain yield, crop water uptake in response to water supply, therefore it can be used to explore strategies for increasing water use efficiencies in NCP [18]. In this paper, the calibrated and validated DSSAT-Wheat model was used to investigate winter wheat growth and soil water balance in response to different irrigation schedules over 22 years. The results of this study were able to determine the quantitative analysis of the winter wheat total productivity in response to changes in water availability for irrigation schedules. The results of this study showed that reduction of irrigation amount will decrease the total productivity. The results were similar to those of Zhao et al. [18]. However, there was a possibility of reducing irrigation amount as well as getting acceptable grain yield and higher WUE with reasonable irrigation schedule.

The preface of this paper indicates that there is a conflict of optimum irrigation schedule. This research showed that intensive irrigation at critical stage was better than multiple irrigation with low amount each time, because the appropriate soil water and higher root density under later conditions was in the surface soil layer [40]. Taking previous researches into consideration, single irrigation should be applied at pre-winter or from recovering to jointing or anthesis of winter wheat. This research also showed that single irrigation at the stages from double ridge to jointing produced higher grain yield than others. Single pre-winter irrigation encouraged root growth at topsoil layer, which restricted water use from deep soil layers [41]. Applying pre-winter irrigation increased soil evaporation from winter to spring. Thus, there was a serious drought stress after anthesis under single pre-winter irrigation. Single irrigation at anthesis cannot alleviate severe drought at spring, which inhibited the tiller emergence. Moreover, dry winds at the end of the growing season can dehydrate wheat very rapidly and reduced the positive effect of applying anthesis irrigation on grain yield. Overall, single irrigation should be applied at early spring growth stages.

Extensive research showed the relationships between grain yield and evapotranspiration. Some research suggested that grain yield increased linearly with evapotranspiration [42]. However, other studies found a quadratic equation between grain yield and evapotranspiration [43–47]. Moreover, Man et al. [48] found that winter wheat grain yield decreased linearly with increasing evapotranspiration. Kang et al. [47] reported that grain yield required a minimum evapotranspiration of 152 mm for winter wheat. The grain yield response to evapotranspiration varied considerably due to differences in soil moisture contents and irrigation scheduling between seasons. This study showed that the relationships between water use efficiency and grain yield or between grain yield and evapotranspiration or transpiration was described by a quadratic equation. And the grain yield increased rapidly as evapotranspiration and transpiration exceeded a certain value. This indicated that obtaining higher yield necessary to maintain a quantity of evapotranspiration and transpiration. Overall, it highlights the importance of supplement irrigation to limited duration and severity of drought stress for attaining relative high yield [49]. This research showed that single irrigation at earlier spring growth stages can obtain high evapotranspiration, and thus it produced higher water use efficiency. Xu et al. [12] reported that single irrigation at earlier spring growth stages could increase soil reservoir capacity to store more accessible rain-water in the soil during the summer season, thereby it increased annual water use.

This study showed that farmer irrigation produced higher income and marginal net return than limiting irrigation and recommending irrigation when there was no water price. Water pricing is a key component of currently agricultural water policy reforms in China, and reasonable pricing reform can improve water management [33]. This study found that the marginal net return of limiting irrigation was equal to that of farmer irrigation at rate of 14.47%, and was equal to that of recommending irrigation at rate of 26.4%. If no subsidies are granted, the farmers would lose money due to the reform. The marginal net return of farmer irrigation and limiting irrigation respectively decrease 762.3 $ ha^-1^ and 290.73 $ ha^-1^ at rate of 26.4%. Zuo [50] reported that the acceptable cost of farmer due to water price reform was 253.27 $ ha^-1^. Liu et al. [51] reported that the subsidy standard was 2.17 times as much as cost due to applying saving irrigation. According to this standard, total marginal net return of irrigation was 1387.5 $ ha^-1^, which is little higher than that of farmer irrigation with no rate.

## Conclusions

Further reduction in irrigation times and amount of water is considered a trade-off to alleviate groundwater overexploitation in NCP. The DSSAT-Wheat model was able to simulate winter wheat development, yield and water balance very well after it was calibrated and validated by the field experimental database which was carried out in NCP. Simulation scenarios of winter wheat response to irrigation scheduling using 22 years of historical weather data showed that obtaining higher yield necessary to maintain a quantity of evapotranspiration and transpiration. Single irrigation at earlier spring growth stages produced a similar water use efficiency compared to recommending irrigation with higher grain yield. Water pricing reform can ensure extension of irrigation strategy in agricultural production. When the water pricing rate was 26.4% maximum value of relative marginal net return, limiting irrigation and recommending irrigation obtained same marginal net return. In order to increase farmer income, there should be subsidy due to applying water-saving irrigation.

## Supporting information

S1 File Effects of irrigation treatments on grain yield, biomass, evapotranspiration and transpiration and soil evaporation over 22 seasons. (PDF)

S2 File (DOCX)
