# Supplementary material for: Evaluation of limited irrigation strategies to improve water use efficiency and wheat yield in the North China Plain
Source: PLoS One. 2018 Jan 25;13(1):e0189989. doi: 10.1371/journal.pone.0189989 (PMC5784901; doi:10.1371/journal.pone.0189989)
Supplement: S1 File — (PDF) [file pone.0189989.s001.pdf]

Table 1. Effect of irrigation on biomass, grain, evapotranspiration (ET), transpiration (T) and soil evaporation (SE) over 22 seasons.

Scenario	Irrigation mm	Biomass kg ha^{-1}	Yield kg ha^{-1}	ET mm	T mm	SE mm
1	140	17581.9 ±1188.6	9021.1 ±845.3	465.7 ±22.2	382.4 ±19.4	79.0 ±12.7
2	60	14201.0 ±1696.2	6874.1 ±914.1	416.0 ±31.6	326.8 ±33.3	84.3 ±14.0
3	90	14952.3 ±1781.2	7726.0 ±963.2	430.8 ±33.6	339.4 ±36.9	86.6 ±14.3
4	90	15778.4 ±1741.2	7851.2 ±1064.2	443.7 ±27.9	357.8 ±26.8	81.6 ±12.9
5	120	16586.1 ±1859.1	8681.3 ±1099.1	458.5 ±28.1	371.3 ±28.4	83.0 ±13.4
6	100	16518.6 ±1141.9	8275.6 ±819.8	450.0 ±24.5	367.3 ±20.6	78.4 ±12.3
7	70	14412.8 ±2465.3	7396.0 ±1597.9	429.2 ±34.2	325.9 ±36.0	98.6 ±10.4
8	70	15847.8 ±1613.0	7966.8 ±1204.6	436.8 ±28.8	353.0 ±25.7	79.5 ±12.0
9	70	15624.0 ±1220.1	7962.1 ±818.1	432.8 ±27.4	349.9 ±23.3	78.5 ±12.2
10	70	13271.9 ±1899.3	6411.5 ±943.1	399.5 ±40.0	302.0 ±47.4	92.1 ±16.4
