# Supplementary material for: Evaluation of limited irrigation strategies to improve water use efficiency and wheat yield in the North China Plain
Source: PLoS One. 2018 Jan 25;13(1):e0189989. doi: 10.1371/journal.pone.0189989 (PMC5784901; doi:10.1371/journal.pone.0189989)
Supplement: S2 File — (DOCX) [file pone.0189989.s002.docx]

Fig 1. Distribution of precipitation, maximum (Tmax) and minimum temperature (Tmin) at Gaocheng, over 22 growth season.





Fig 2. Comparisons of measured and observed above-ground biomass (a for 2013-2014 and b for 2014-2015) and LAI (c for 2013-2014 and d for 2014-2015). a and c were used to calibrate the model, b and d were used to validate the model.





Fig 3. Validation results of the CERES-wheat model for evapotranspiration (a) and grain yield (b).





Fig 4. Simulated grain and biomass yield for different irrigation treatments. The triangle indicates the median. The circle indicates the 75 and 25 percent. The middle line indicates 50 percent.





Fig 5. Simulated evapotranspiration (ET) and water use efficiency (WUE) for different treatments. The triangle indicates the median. The circle indicates the 75 and 25 percent. The middle line indicates 50 percent.





Fig 6. Grain yield response to evapotranspiration (ET) and transpiration and soil evaporation over 22 years.





Fig 7. Simulated marginal net return (MNR), relative marginal net return (RMNR) and net water use (NWU) for different irrigation over 22 year seasons.





Fig 8. Simulated net return for different irrigation strategies over 22 seasons.

**Table 1. Water and nitrogen treatments in the experiments conducted at Gaocheng, China (2013-2015).**

|  | Total irrigation amount (mm) | Jointing Irrigation (mm) | Anthesis Irrigation (mm) | Grain Filling Irrigation (mm) | N application (N kg ha^-1^) |
| --- | --- | --- | --- | --- | --- |
| Experiment 1 | 200 | 100 | 100 | 0 | 240 |
|  | 120 | 60 | 60 | 0 | 195 |
|  | 105 | 45 | 30 | 30 | 195 |
| Experiment 2 | 67.5 | 67.5 | 0 | 0 | 120, 180, 240 |
|  | 67.5 | 37.5 | 30 | 0 | 120, 180, 240 |
|  | 67.5 | 37.5 | 15 | 15 | 120, 180, 240 |
|  | 135 | 67.5 | 67.5 | 0 | 120, 180, 240 |

**Table 2. Genetic coefficients calibrated for the 2013-2014 and 2014-2015 seasons.**

| Genetic coefficient | Definition | Calibrated values |
| --- | --- | --- |
| P1V | Days, optimum vernalizing temperature, required for vernalization | 45 |
| P1D | Photoperiod response (% reduction in rate/10 h drop in pp) | 68 |
| P5 | Grain filling (excluding lag) phase duration (℃ day) | 800 |
| G1 | Kernel number per unit canopy weight at anthesis (g) | 31 |
| G2 | Standard kernel size under optimum conditions (mg) | 35 |
| G3 | Standard, non-stressed mature tiller weight (including grain) (g dry weight) | 1.4 |
| PHINT | Interval between successive leaf tip appearances (℃ day) | 100 |

**Table 3. Irrigation schedules simulated with the CERES-Wheat model.**

| Treatment | Sowing, mm | Double ridge, mm | Jointing, mm | Anthesis, mm | Grain filling, mm | Total, mm |
| --- | --- | --- | --- | --- | --- | --- |
| T1 |  |  | 70 | 70 |  | 140 |
| T2 |  |  | 30 | 30 |  | 60 |
| T3 |  |  | 30 | 30 | 30 | 90 |
| T4 |  | 30 | 30 | 30 |  | 90 |
| T5 |  | 30 | 30 | 30 | 30 | 120 |
| T6 |  |  | 70 | 30 |  | 100 |
| T7 | 70 |  |  |  |  | 70 |
| T8 |  | 70 |  |  |  | 70 |
| T9 |  |  | 70 |  |  | 70 |
| T10 |  |  |  | 70 |  | 70 |

**Table 4. Economic costs for different irrigation regimes over 22 seasons.**

| Irrigation regime | Irrigation  amount  mm | Yield  kg ha^-1^ | Cost  $ ha^-1^ | Income  $ ha^-1^ | MNR  $ ha^-1^ | RMNR  $ ha^-1^ mm^-1^ | NWU  mm |
| --- | --- | --- | --- | --- | --- | --- | --- |
| Rainfed | 0 | 5280.6 | 673 | 1228.0 |  |  | 29.4 |
| Limiting irrigation | 80 | 8244.3 | 753 | 2214.9 | 986.9 | 12.34 | -50.6 |
| Recommending irrigation | 140 | 9021.1 | 815 | 2432.6 | 1204.6 | 8.60 | -110.6 |
| Farmer irrigation | 210 | 9520.9 | 894 | 2533.5 | 1305.5 | 6.21 | -210.6 |

US$=6.6 Chinese Yuan. The prices for land preparing and sowing were: water power ($0.08 m^-3^), plough ($68 ha^-1^), rotary ($45 ha^-1^), sowing wheat ($56 ha^-1^), wheat seed ($0.52 kg^-1^), herbicides and pesticides ($23 ha^-1^), harvest ($145 ha^-1^). Fertilizer prices: N ($0.59 kg^-1^), P_2_O_5_ ($0.52 kg^-1^). Labor price: $8 day^-1^. Wheat grain: $0.36 kg^-1^.
